# Reply letter to comments on: Targeting the HGF/c-MET pathway in advanced pancreatic cancer: a key element of treatment that limits primary tumour growth and eliminates metastasis

**DOI:** 10.1038/s41416-020-1004-6

**Published:** 2020-07-28

**Authors:** Zhihong Xu, Srinivasa Pothula, David Goldstein, Romano C. Pirola, Jeremy S. Wilson, Minoti V. Apte

**Affiliations:** 1 Pancreatic Research Group, South Western Sydney Clinical School, Faculty of Medicine, UNSW, Sydney, NSW Australia; 2 Ingham Institute for Applied Medical Research, Sydney, NSW Australia

**Keywords:** Cancer microenvironment, Targeted therapies

We thank Che and colleagues for their interest in our recently published study. We were pleased to see that the authors agree with our concept that targeting both cancer cells and pancreatic stellate cells is of crucial importance in interrupting stromal–tumour interactions and improving outcomes in pancreatic cancer. The authors have made some reasonable suggestions for future work in this area with regard to gemcitabine resistance, differential c-MET expression and combination with other treatment modalities.

With regard to the issue of collagen content of tumours, as noted by Che et al., a significant decrease in collagen staining in tumours was observed only when both HGF and c-MET were inhibited, while the addition of gemcitabine to this treatment resulted in collagen expression being reversed to control levels. Interestingly, as shown in Supplementary Fig. 1b,^1^ gemcitabine alone did increase collagen content of tumours compared with the control (untreated) group (*P* < 0.02, Student’s *t* test). The same trend of elevated collagen deposition in gemcitabine versus control groups has also been shown in our earlier studies.^2,3^ It is possible therefore that in the triple-treatment group (HGF/c-MET inhibition + gemcitabine), the reduction in collagen by HGF/c-MET inhibition was negated by the opposing effects of gemcitabine. While cancer cells may have the capacity to participate in collagen deposition, it is widely acknowledged that the predominant source of collagen in pancreatic cancer are pancreatic stellate cells. We speculate that in the gemcitabine-treated group, the cytotoxic effects of the drug lead to the release of chemokines, cytokines and other pro-fibrogenic factors from the dying cancer cells, which could stimulate extracellular matrix synthesis by pancreatic stellate cells. Nonetheless, we agree with Che and colleagues that the role of the extracellular matrix in pancreatic cancer progression is an area worthy of further investigation.

## Data Availability

All data and materials are published in this letter or available on request.
